# Diagnostic performance of chest CT in differentiating COVID-19 from other causes of ground-glass opacities

**DOI:** 10.1186/s43055-020-00398-6

**Published:** 2021-01-05

**Authors:** Ali H. Elmokadem, Dalia Bayoumi, Sherif A. Abo-Hedibah, Ahmed El-Morsy

**Affiliations:** 1 Department of Radiology, Mansoura University, Mansoura, Egypt; 2 Department of Radiology, Farwaniya Hospital, Sabah Al Nasser, Kuwait; 3 Department of Radiology, Cairo University, Giza, Egypt

**Keywords:** COVID-19, SARS-COV2, Computed tomography, Diagnosis, Ground-glass opacity

## Abstract

**Background:**

To evaluate the diagnostic performance of chest CT in differentiating coronavirus disease 2019 (COVID-19) and non-COVID-19 causes of ground-glass opacities (GGO).

**Results:**

A total of 80 patients (49 males and 31 females, 46.48 ± 16.09 years) confirmed with COVID-19 by RT-PCR and who underwent chest CT scan within 2 weeks of symptoms, and 100 patients (55 males and 45 females, 48.94 ± 18.97 years) presented with GGO on chest CT were enrolled in the study. Three radiologists reviewed all CT chest exams after removal of all identifying data from the images. They expressed the result as positive or negative for COVID-19 and recorded the other pulmonary CT features with mention of laterality, lobar affection, and distribution pattern. The clinical data and laboratory findings were recorded. Chest CT offered diagnostic accuracy ranging from 59 to 77.2% in differentiating COVID-19- from non-COVID-19-associated GGO with sensitivity from 76.25 to 90% and specificity from 45 to 67%. The specificity was lower when differentiating COVID-19 from non-COVID-19 viral pneumonias (30.5–61.1%) and higher (53.1–70.3%) after exclusion of viral pneumonia from the non-COVID-19 group. Patients with COVID-19 were more likely to have lesions in lower lobes (*p* = 0.005), peripheral distribution (*p* < 0.001), isolated ground-glass opacity (*p* = 0.043), subpleural bands (*p* = 0.048), reverse halo sign (*p* = 0.005), and vascular thickening (*p* = 0.013) but less likely to have pulmonary nodules (*p* < 0.001), traction bronchiectasis (*p* = 0.005), pleural effusion (*p* < 0.001), and lymphadenopathy (*p* < 0.001).

**Conclusions:**

Chest CT offered reasonable sensitivity when differentiating COVID-19- from non-COVID-19-associated GGO with low specificity when differentiating COVID-19 from other viral pneumonias and moderate specificity when differentiating COVID-19 from other causes of GGO.

## Background

The coronavirus disease outbreaks reaches up to more than 15 million positive cases and more than 600 thousand deaths among 216 affected countries, areas, and territories as recorded by WHO in July 2020 [1]. Although starting in China, the USA scored more than 50% of these recorded positive cases. Patients infected with coronavirus disease 2019 (COVID-19) present with fever, cough, dyspnea, and muscle aches [2]. The gold standard for diagnosis is PCR for oropharyngeal swab, nasopharyngeal swab, bronchoalveolar lavage, or tracheal aspirate [3]. However, recently, reverse transcription-polymerase chain reaction test (RT-PCR) shows relative low sensitivity at 60–71% for detecting COVID-19 [2, 4, 5], which can be explained by the lower viral overload in swap or laboratory error [4, 6]. On the other hand, chest CT has demonstrated about 56–98% sensitivity in detecting COVID-19 at early stages of the disease [4, 5]; nevertheless, chest CT shows low specificity (25%) for COVID-19 diagnosis as reported in recent studies [2].

The typical chest CT findings for of COVID-19 pneumonia are multifocal ground-glass opacity (GGO) of rounded morphology with characteristic bilateral peripheral distribution that can be associated with consolidation and crazy-paving patterns [7]. Vascular dilatation and traction bronchiectasis are also typical findings found in the GGO detected in COVID-19 patients [8]. Architectural distortion with the formation of subpleural bands was reported in some cases during a peak stage of the disease [9]. Indeterminate features of COVID-19 include multifocal, diffuse, perihilar, or unilateral GGO with or without consolidation, non-specific distribution, or non-rounded GGO [5]. Other findings typically were seen in infection as thickening of the bronchial wall, mucoid impactions, and centrilobular nodules (tree-in-bud), while lymphadenopathy and pleural effusion are rarely observed [7, 10].

A recent meta-analysis assessed the performance of RT-PCR and chest CT in diagnosis of COVID-19 [11]; the pooled sensitivity for RT-PCR was 89% (95% CI: 81%, 94%; *I*^2^ = 90%) and that for chest CT was higher reaching up to 94% (95% CI: 91%, 96%; *I*^2^ = 95%), yet the pooled specificity of chest CT was low as 35% (95% CI:26%, 50%; *I*^2^ = 95%). The positive predictive value (PPV) for RT-PCR ranged from 47.3 to 96.4% and the negative predictive value (NPV) ranged from 96.8 to 99.9% while the PPV for CT ranged from 1.5 to 30.7% and NPV ranged from 95.4 to 99.8%. Given the large gap found between PPV of chest CT vs RT-PCR and low specificity of CT, the use of chest CT may result in a large percentage of false-positive results that may cause extra diagnostic investigation and higher medical cost, hospital load, and patient uneasiness.

The low specificity of chest CT may be attributed to the presence of a wide range of pulmonary conditions that can mimic the CT appearance of COVID-19 especially the ones associated with GGO. The commonest causes of the GGO that can simulate COVID-19 are viral pneumonia, atypical bacterial pneumonia, *Pneumocystis jiroveci* pneumonia (PJP), interstitial pneumonia, hypersensitivity pneumonitis, eosinophilic pneumonia, diffuse alveolar hemorrhage, drug-induced lung injury, and pulmonary edema (cardiogenic and non-cardiogenic). Therefore, the purpose of this study is to assess the diagnostic performance of chest CT in differentiating COVID-19 and non-COVID-19 causes of GGO.

## Methods

This retrospective study was approved a by local institutional review board, and a waiver of consent of medical record review was received. The study included 180 adult patients who underwent non-contrast CT study of the chest during the period from April 2019 and June 2020. The first group comprised of 80 patients (49 males and 31 females, 46.48 ± 16.09 years) who were selected after a positive RT-PCR test for COVID-19 and positive CT findings within 14 days after the swap result. The second group consisted of 100 patients (55 males and 45 females, 48.94 ± 18.97 years) who have GGO secondary to causes rather than COVID-19 disease and were selected using a search engine for ground-glass opacities among CT reports on hospital Picture Archiving and Communication system (PACS). Patients’ clinical data were extracted from medical records. Radiologic, bronchoscopic, and pathologic reports were reviewed by one author (AHE) to identify the culprit pathologies for the second group. We only included cases that were proved by PCR, bronchoalveolar lavage (BAL), sputum culture, blood testing, or follow-up after proper treatment.

Spiral CT scan was done for all patients from the root of the neck to the level of the upper pole of the kidneys during a single-breath hold using 1 mm slice thickness. Images were reconstructed in axial, coronal, and sagittal reformats with standard pulmonary filtering.

Three radiologists with more than 10 years of experience (AE, DB, and SAA) were blinded from the final diagnoses and PCR findings and reviewed all CT chest exams after removal of all identifying data from the images. They expressed the result as positive or negative for COVID-19 based on the Radiological Society of North America expert consensus [12]. The consensus reports chest CT findings attributed to COVID-19 into four categories: (i) typical COVID-19 that displays bilateral, peripheral, or multifocal rounded GGO of rounded morphology with or without consolidation, “crazy-paving” pattern, or reversed halo sign; (ii) indeterminate COVID-19 that manifests as multifocal, diffuse, perihilar, or unilateral GGO with or without consolidation, non-specific distribution, or non-rounded GGO; (iii) atypical COVID-19 with atypical CT features such as lobar or segmental consolidation without GGO, pulmonary nodules (centrilobular or “tree in-bud”), pulmonary cavitation, smooth interlobular septal thickening, pleural effusion, and lymphadenopathy; and (iv) negative from pneumonia. Readers were asked to give positive results in case of typical category and negative results in case of atypical and negative categories while cases with indeterminate features were left for the reader to decide based on typical versus indeterminate features in each case.

Additionally, they were asked to assess the presence of other CT findings associated with COVID-19 such as consolidations, crazy-paving, subpleural bands, vascular dilatation, and reverse halo sign as well as atypical features of COVID-19 such as pulmonary nodules (centrilobular or tree-in-bud), mediastinal lymphadenopathy, and pleural effusion with mention of laterality, lobar affection (upper or lower), and distribution pattern (peripheral, central, or diffuse).

Continuous variables were expressed as medians and ranges while categorical variables were expressed as numbers and percentages. To assess the radiologists’ diagnostic efficiency, metrics such as sensitivity, specificity, positive predictive value, negative predictive value, and accuracy were measured. COVID-19 was considered a positive finding for the results, while other etiology was pneumonia, and neither was considered a negative result. Exact binomial 95% confidence intervals were calculated for sensitivity, specificity, PPV, NPV, and accuracy using Statistical Package for Social Science version 20 (SPSS Inc., Chicago, IL, USA). *P* values of < 0.05 were considered statistically significant.

## Results

Our final cohort consisted of 80 patients with COVID-19- and 100 patients with non-COVID-19-associated GGO. There was no significant statistical difference as regards the age (*p* value = 0.131) between both groups. Non-COVID-19 cases were secondary to viral pneumonia in 36 patients, atypical bacterial pneumonia in 8 patients, PJP in 6 patients, interstitial pneumonias in 15 patients, hypersensitivity pneumonia in 8 patients, eosinophilia pneumonia in 4 patients, pulmonary alveolar hemorrhage in 6 patients, drug-induced lung injury in 7 patients, and pulmonary edema (cardiogenic and non-cardiogenic) in 10 patients. Table 1 shows the etiologies of non-COVID-19 GGO, the different viral and bacterial pathogens, and types of interstitial pneumonia.

**Table 1:** Etiology of ground-glass opacities in study groups

COVID-19	80
Viral pneumonia	36
Influenza A (H1N1)	15
SARS	4
MERS	2
RSV	3
HSV	4
Adenovirus	2
Rhinovirus	6
Atypical bacterial pneumonias	8
Mycoplasma	4
Chlamydia	3
Klebsiella	1
PJP	6
Interstitial pneumonias	15
NSIP	6
DIP	3
COP	6
HP	8
EP	4
DAH	6
Drug-induced lung injury	7
Pulmonary edema (cardiogenic and non-cardiogenic)	10

*SARS* sever acute respiratory syndrome, *MERS* Middle East respiratory syndrome, *RSV* respiratory syncytial virus, *HSV* herpes simplex virus, *PJP Pneumocystis jiroveci* pneumonia, *NSIP* non-specific interstitial pneumonia, *DIP* desquamative interstitial pneumonia, *COP* cryptogenic interstitial pneumonia, *HP* hypersensitivity pneumonia, *EP* eosinophilia pneumonia, *DAH* diffuse alveolar hemorrhage

Compared to non-COVID-19 patients, COVID-19 patients have more fever (90% vs. 62%, *p* < 0.001) and have gastrointestinal manifestations such as diarrhea, nausea, and vomiting (18.75% vs. 5%, *p* < 0.001). There were no significant differences as regards respiratory symptoms (cough and dyspnea) between both groups (*p* = 0.218). Lymphopenia was found more common in COVID-19 patients (55% vs. 21%, *p* < 0.001) while leukocytosis was found more common in non-COVID-19 patients (15% vs. 56%, *p* < 0.001).

**Fig. 1:**
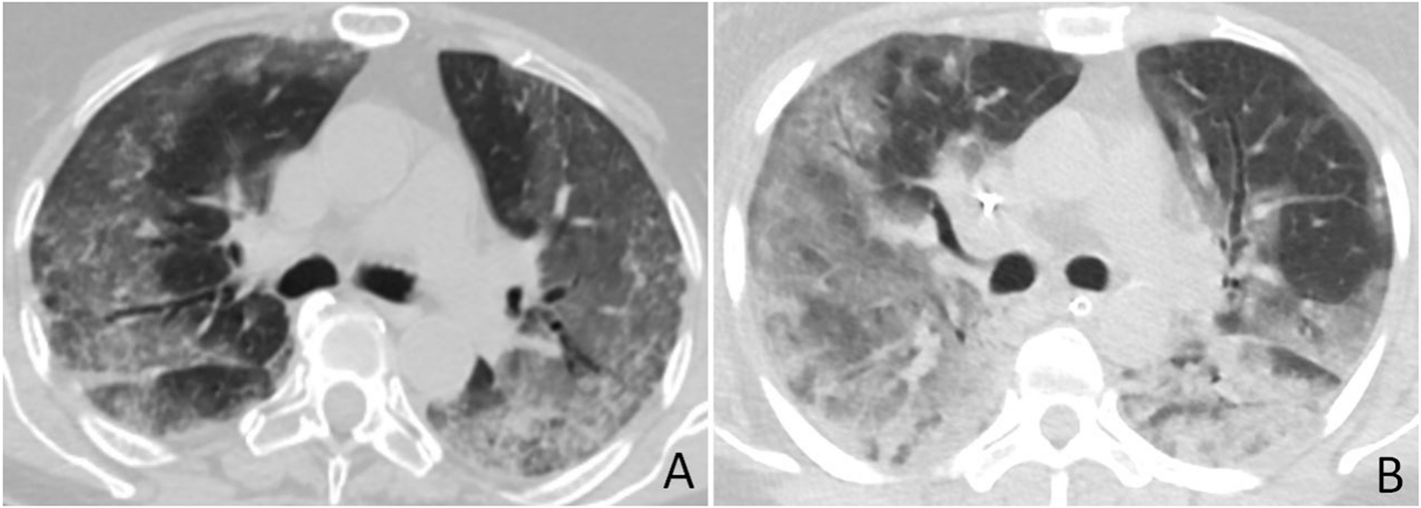
COVID-19 and MERS pneumonia misdiagnosed as COVID-19. **a** A 69-year-old man with positive PCR test for COVID-19; axial HRCT image shows peripheral and central GGO with superimposed interlobular septal thickening, bronchial dilatation, and pleural thickening. **b** Axial CT image of a 50-year-old man with a positive PCR for MERS shows similar findings as **a** with posteriorly located consolidations in the right lung

**Fig. 2:**
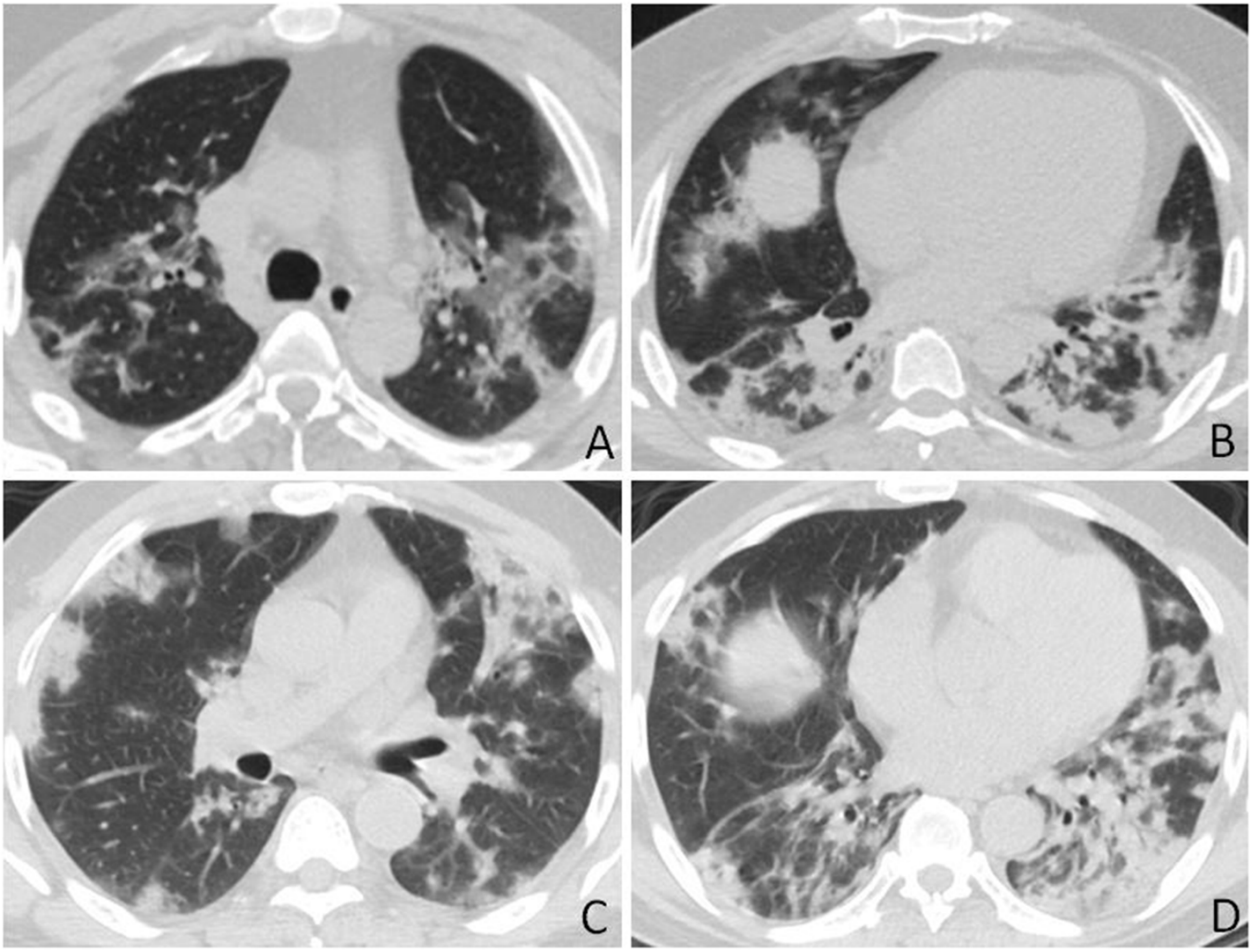
COVID-19 and influenza A pneumonia misdiagnosed as COVID-19. **a** and **b** Axial CT images obtained from a 43-year-old woman with positive PCR test for COVID-19 show peripherally located multifocal areas of poorly defined focal consolidation, small areas of GGO, and bronchial wall thickening. **c** and **d** Axial CT images from a 61-year-old man with positive PCR test for influenza A (H1N1) show similar features as **a** and **b** with predominant consolidations

**Fig. 3:**
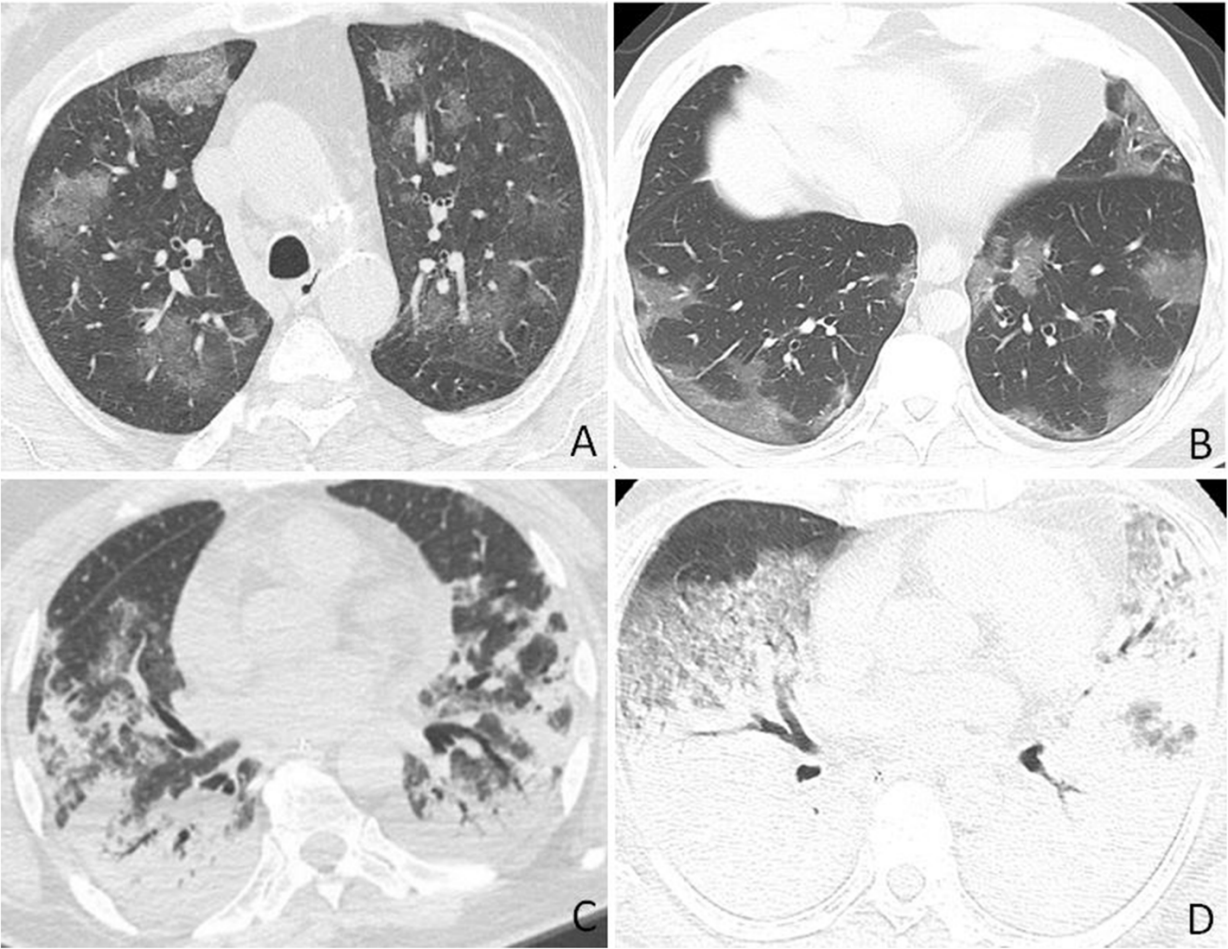
COVID-19 and bacterial pneumonia misdiagnosed as COVID-19. **a** Axial CT image obtained from a 49-year-old man with positive PCR test for COVID-19 shows peripherally located multifocal areas of GGO and superimposed interlobular septal thickening. **b** Axial CT image obtained for a 38-year-old man with Klebsiella pneumonia shows peripherally located GGO as shown in (**a**). **c** Axial CT image obtained from a 67-year-old woman admitted to ICU with positive PCR for COVID-19 and secondary infection by *Staphylococcus aureus*; axial CT shows bilateral consolidation with small areas of GGO and bronchial dilatation. **d** Axial CT image from a 72-year-old woman with MRSA pneumonia (Methicillin-resistant *Staphylococcus aureus*) shows bilateral larger consolidations and GGO than the ones seen on (**c**)

**Fig. 4:**
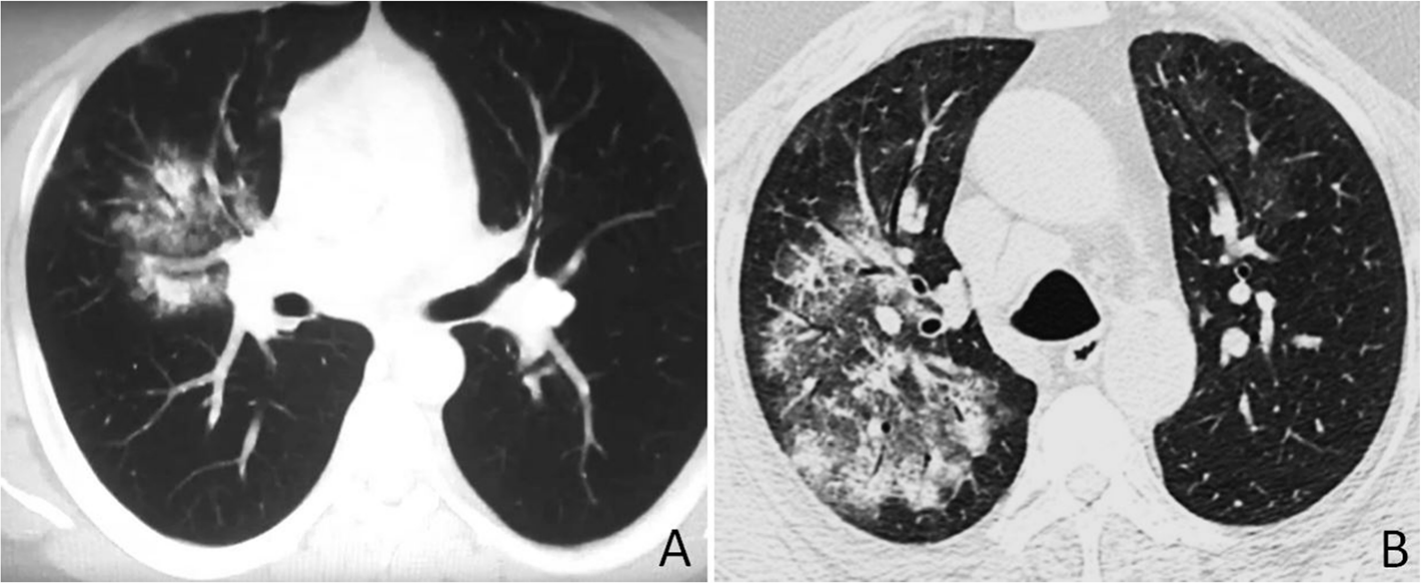
COVID-19 and cryptogenic pneumonia misdiagnosed as COVID-19. **a** Axial CT image obtained from a 33-year-old man with positive PCR test for COVID-19 shows area of clearing consolidation with central ground-glass density “reversed halo sign” in the right lung. **b** Cryptogenic organizing pneumonia in a 55-year-old man with history of chest infection not responding to multiple courses of antibiotics; axial CT image shows reversed halo sign in the right lung with small areas of GGO in the left lung. Transbronchial biopsy showed findings of COP

**Fig. 5:**
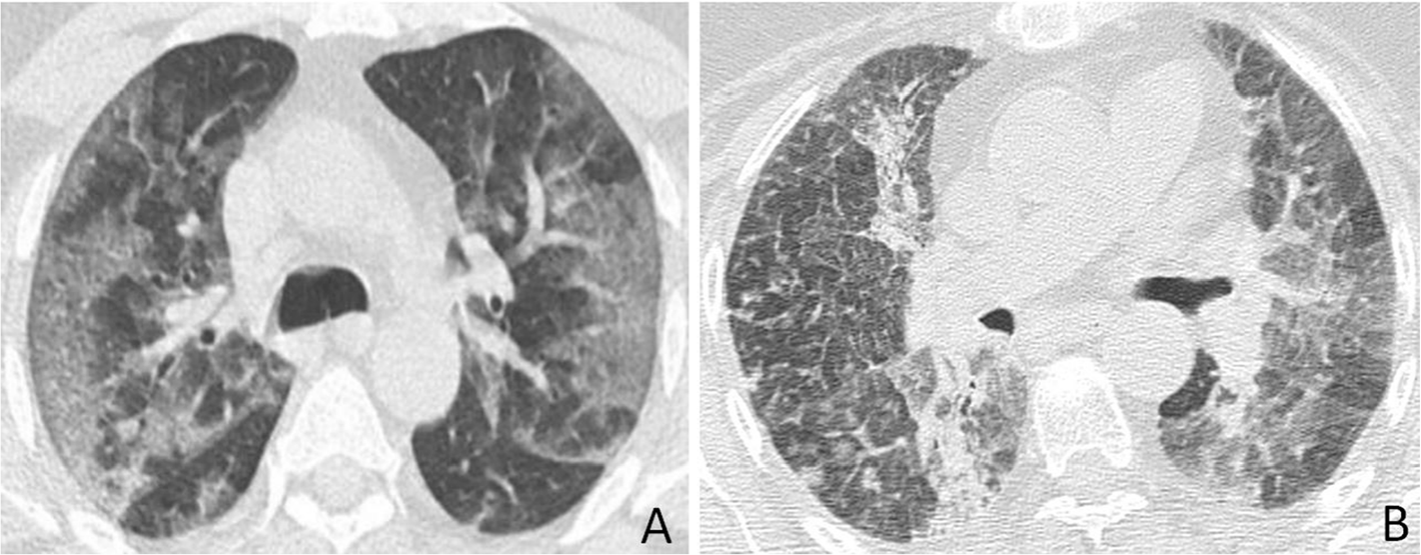
COVID-19 and drug-induced lung injury misdiagnosed as COVID-19. **a** Axial CT image obtained from a 62-year-old man with positive PCR test for COVID-19 shows multifocal peripheral GGO with superimposed interlobular septal thickening and visible intralobular lines (“crazy-paving”). **b** Bleomycin-induced lung injury in a 55-year-old woman with history of non-Hodgkin lymphoma; axial CT image shows multifocal areas of GGO, consolidations, and few pulmonary nodules in the right lung

**Fig. 6:**
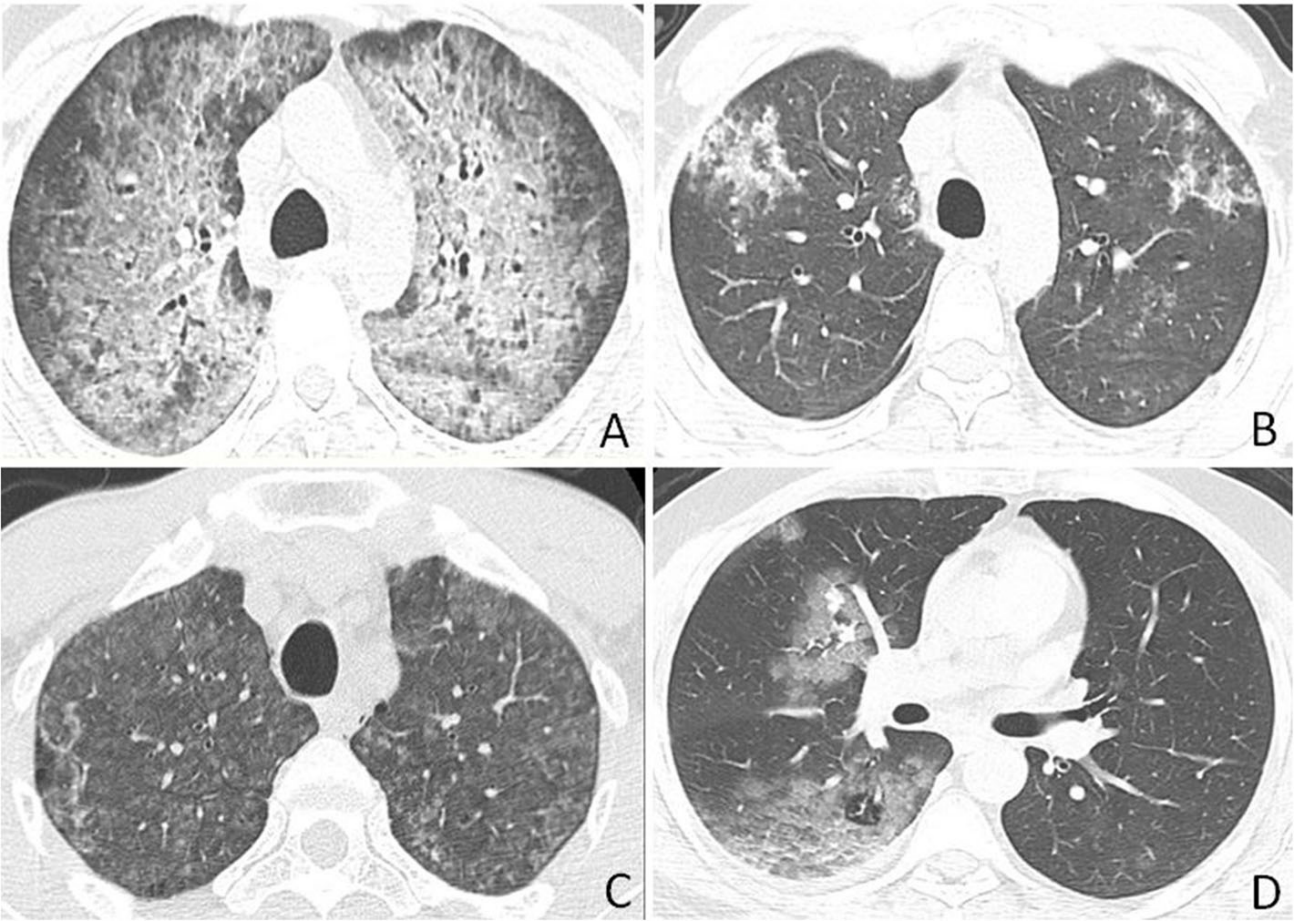
Examples of conditions that were not mistaken as COVID-19. **a** *Pneumocystis jiroveci* pneumonia in a 33-year-old man with positive HIV test; axial CT image shows extensive bilateral ground-glass opacities with relative subpleural sparing. **b** Rhinovirius pneumonia in a 42-year-old woman; axial CT image shows multiple ill-defined patchy areas of GGO with left pulmonary nodules. **c** A 31-year-old farmer with subacute hypersensitivity pneumonitis; axial CT image shows patchy or diffuse bilateral ground-glass opacities associated with poorly defined centrilobular nodules. **d** A 66-year-old man with diffuse alveolar hemorrhage; axial CT image shows asymmetric bilateral patchy areas of GGO

Patients with COVID-19 were more likely to have isolated ground-glass opacity (21.5% vs. 13%, *p* = 0.043), subpleural bands (30% vs. 19%, *p* = 0.048), reverse halo sign (11.25% vs. 3%, *p* = 0.005), and vascular thickening (36.25% vs. 17%, *p* = 0.013) but less likely to have pulmonary nodules (21.5% vs. 41%, *p* < 0.001), traction bronchiectasis (16.25 vs. 39%, *p* = 0.005), pleural effusion (5 vs. 32%, *p* < 0.001), and lymphadenopathy (0% vs. 12%, *p* < 0.001). Compared to non-COVID-19 patients, COVID-19 patients have more lesions in lower lobes (88.75% vs. 65%, *p* = 0.005) and peripheral distribution (78.75% vs. 47%, *p* < 0.001). There were no significant differences between presence of consolidation, crazy-paving pattern, and laterality on both groups. Patients’ demographics, clinical, laboratory data, and imaging features of both groups are demonstrated in Table 2. Figures 1, 2, 3, 4 and 5 demonstrate example cases in which diagnosis was overlapped between both groups. Figure 6 shows example of conditions that were not mistaken as COVID-19.

**Table 2:** Patients’ demographics, clinical characteristics, laboratory data, and imaging features of both groups

	COVID-19 (*n* = 80)	Non-COVID-19 (*n* = 100)	*P* value
Age	46.48 ± 16.09	48.94 ± 18.97	0.131
Sex			
Male	49 (61.25%)	55 (55%)	0.108
Female	31 (38.75%)	45 (45%)	0.098
Clinical data			
Fever	72 (90%)	62 (62%)	< 0.001
Cough and dyspnea	65 (81.25%)	85 (85%)	0.218
GIT manifestations	15 (18.75%)	5 (5%)	< 0.001
Laboratory findings			
Leukocytosis	12 (15%)	56 (56%)	< 0.001
Lymphopenia	44 (55%)	21 (21%)	< 0.001
CT features			
Isolated GGO	17 (21.5%)	13 (13%)	0.043
Consolidation	54 (65%)	72 (72%)	0.268
Crazy-paving	15 (18.75%)	21 (21%)	0.369
Reversed halo	9 (11.25%)	3 (3%)	0.005
Subpleural bands	24 (30%)	19 (19%)	0.048
Vascular thickening	29 (36.25%)	17 (17%)	0.013
Traction bronchiectasis	13 (16.25%)	39 (39%)	0.005
Pulmonary nodules	17 (21.5%)	41 (41%)	< 0.001
Lymphadenopathy	0 (0%)	12 (12%)	< 0.001
Pleural effusion	4 (5%)	32 (32%)	< 0.001
Laterality			
Unilateral	12 (15%)	19 (19%)	0.323
Bilateral	68 (85%)	81 (81%)	0.297
Lobar affection			
Upper	43 (53.75%)	67 (67%)	0.039
Lower	71 (88.75%)	65 (65%)	0.005
Distribution			
Peripheral	63 (78.75%)	46 (46%)	< 0.001
Central	2 (2.5%)	13 (13%)	< 0.001
Diffuse	15 (18.75%)	41 (41%)	< 0.001

For all chest CTs, three radiologists correctly differentiated COVID-19 from other causes of GGO with degree of accuracy 69.4% (125/180), 77.2% (139/180), and 59% (106/180). The sensitivity was 86.25% (95 CI: 77–93%), 90% (95 CI: 81–95%), and 76.25% (95 CI: 65–85%) while specificity was 56% (95 CI: 46–66%), 67% (95 CI: 57–76%), and 45% (95 CI: 35–50%). Table 3 shows the performance results of the three radiologists for differentiation between COVID-19 and non-COVID-19 associated GGO. In a subgroup analysis, we analyzed the diagnostic performance of chest CT to differentiate COVID-19 from non-COVID-19 viral pneumonias. The specificity was lower to differentiate COVID-19 from non-COVID-19 viral pneumonias (30.5–61.1%), and subsequently, specificity was higher (53.1–70.3%) after exclusion of viral pneumonia from non-COVID-19 group. Tables 4 and 5 show performance results of the three radiologists for differentiation between COVID-19 and non-COVID-19 viral pneumonias and non-COVID-19 group after exclusion of viral pneumonias.

**Table 3:** Performance results of the three radiologists for differentiation between COVID-19- and non-COVID-19-associated GGO

	TP	TN	FP	FN	Sensitivity (95% CI)	Specificity (95% CI)	PPV (95% CI)	NPV (95% CI)	Accuracy (95% CI)
Radiologist 1	69	56	44	11	86.25% (77–93%)	56% (46–66%)	61.1% (55–66%)	83.6% (74–90%)	69.4% (62%–76%)
Radiologist 2	72	67	33	8	90% (81–95%)	67% (57–76%)	68.6% (62–74%)	89.3% (81–94%)	77.2% (70–83%)
Radiologist 3	61	45	55	19	76.25% (65–85%)	45% (35–55%)	52.6% (47–58%)	70.3% (60–79%)	59% (51–66%)

**Table 4:** Performance results of the three radiologists for differentiation between COVID-19 and non-COVID-19 viral pneumonias

	TP	TN	FP	FN	Sensitivity (95% CI)	Specificity (95% CI)	PPV (95% CI)	NPV (95% CI)	Accuracy (95% CI)
Radiologist 1	69	16	20	11	86.25% (77–93%)	44.4% (28–62%)	77.5% (72–82%)	59.3% (43–74%)	73.3% (64–81%)
Radiologist 2	72	22	14	8	90% (81–95%)	61.1% (43–77%)	83.7% (77–88.6%)	73.3% (57–85%)	81% (73–88%)
Radiologist 3	61	11	25	19	76.25% (65–85%)	30.5% (16–48%)	70.1% (65–76%)	36.7% (23–52%)	62% (52–71%)

**Table 5:** Performance results of the three radiologists for differentiation between COVID-19- and non-COVID-19-associated GGO after exclusion of viral pneumonias

	TP	TN	FP	FN	Sensitivity (95% CI)	Specificity (95% CI)	PPV (95% CI)	NPV (95% CI)	Accuracy (95% CI)
Radiologist 1	69	40	24	11	86.25% (77–93%)	62.5% (49–74%)	74.2% (67–80%)	78.4% (67–87%)	75.7% (68–82%)
Radiologist 2	72	45	19	8	90% (81–95%)	70.3% (57–81%)	79.1% (72–85%)	84.9% (74–92%)	81.2% (74–87%)
Radiologist 3	61	34	30	19	76.25% (65–85%)	53.1% (40–66%)	67% (60–73%)	64.1% (53–74%)	65.9% (58–74%)

## Discussion

The sudden outbreak of the novel coronavirus disease (COVID-19) late in 2019 raised serious public health concerns due to its rapid human to human transmission with the possibility of it causing fatal ARDS. The initial standard diagnostic method was RT-PCR through pharyngeal swabs which had high sensitivity but low specificity (60–70%) in detecting the viral RNA resulting in a large number of false-negative results which required repeated testing and added more strain to the medical infrastructures, not to mention it was time-consuming and relatively expensive [13]. To overcome these drawbacks, HRCT chest was suggested as an additional method of diagnosis allowing rapid detection of the disease and helping quarantine of COVID-19-suspected cases and their contacts [14].

In this study, chest CT offered reasonable sensitivity ranging from 76.25 to 90% in differentiating COVID-19- from non-COVID-19-associated GGO with resultant diagnostic accuracy ranging from 59 to 77.2%. But the specificity was low to moderate ranging from 45 to 67%, because of the similarity between the radiological appearance of COVID-19 pneumonia and other viral infections. A recent study conducted by Bai et al. [9] included 424 chest CT exams from the USA and China and compared the diagnostic accuracy of two different teams of radiologists from both countries. The accuracy for the Chinese radiologists ranged from 60 to 83%, the sensitivity was 72 to 94%, while the specificity was extremely variable ranging from 24 to 94%. The accuracy for American radiologists was higher ranging from 83 to 97%. The sensitivity was 93, 83, 73, and 70% while the specificity was 100, 93, 93, and 100%. Li et al., 2020, conducted a similar larger-sized retrospective multicentric study [15], to differentiate COVID-19 disease from community-acquired pneumonia (CAP) and other non-pneumonia abnormalities. The sensitivity and specificity for detecting COVID-19 were 90% (95% CI: 83%, 94%; *p* < 0.001) and 96% (95% CI: 93%, 98%; *p* < 0.001), respectively. While for detection of CAP, the sensitivity and specificity were 87% (95% CI: 81%, 91%; *p* < 0.001) and 92% (95% CI, 88%, 95%; *p* < 0.001). They got better results when they took the advantages of an AI system that was fed with the data of 4352 chest CT exams from 3322 patients. Another study also conducted by Bai et al. [16] evaluated the performance of radiologists in the identification of COVID pneumonia without and with AI technology assistance, and they reported that the AI system helped the radiologists to achieve higher diagnostic performance with average diagnostic accuracy (90% vs. 85%, *p* < 0.001), sensitivity (88% vs. 79%, *p* < 0.001), and specificity (91% vs. 88%, *p* = 0.001).

The specificity in the current study was the lowest 30.5, 44.4, and 61.1% when we compared the performance of the three radiologists for differentiating COVID-19 and non-COVID-19 viral pneumonia because of the resemblance between the two categories, although certain radiological features were more common in COVID-19 such as peripherally distributed GGO with lower lobes predominance, subpleural bands, vascular thickening, and reversed halo sign. In contrast, lesions in non-COVID-19 group showed central and peripheral distribution with higher incidence of pulmonary nodules, traction bronchiectasis, pleural effusion, and lymphadenopathy. The situations where our radiologists encountered difficulties in differentiating the COVID-19 disease from other diseases with confidence were when the COVID-19 disease was atypical, or when the condition was complicated by bacterial infection or associated with a previously unreported chest condition.

Other respiratory viruses such as influenza virus show a lesser incidence of rounded GGO and interstitial thickening with more common diffuse GGO, nodular densities, tree-in-bud appearance, and pleural effusion [17]. More severe unifocal lung involvement including GGO, pulmonary consolidation, air-bronchogram pattern, and septal thickening with absent pulmonary nodules and reversed halo sign is seen in other coronavirus diseases including severe acute respiratory syndrome (SARS) and Middle East respiratory syndrome (MERS) [18]. In the late stages of viral infections such as HIV, CMV, and HPV especially in old ages, organ transplantation, and immune-compromised patients, there are patchy, multifocal widely distributed GGO and consolidations with pleural effusion resulting in ARDS [19]. Nevertheless, the specificity improved for our three radiologists reaching 53.1, 62.5, and 70.3% after exclusion of viral pneumonia from the non-COVID-19 group. The other causes of GGO are a heterogeneous group of diseases with a lesser degree of resemblance with COVID-19.

In contrast to COVID-19, bacterial pneumonia causes segmental pulmonary opacities without specific site predominance. Frequently, it is associated with lung abscesses, lymphadenopathy, effusions, or empyema. Sometimes COVID-19 patients may experience secondary bacterial infection which makes it more difficult to diagnose and treat [20]. Unlike COVID-19, pneumocystis pneumonia have presents with pulmonary nodules, cysts, and pneumothorax with slight upper lobe predominance but in advanced cases (immunocompromised and HIV patients), it results in diffuse GGO, consolidations and crazy-paving pattern [21].

Most of interstitial lung pneumonia has an insidious onset in contrary to acute presentation of COVID-19. Non-specific interstitial pneumonia (NSIP) is usually predisposed by connective tissue disorders and presented on CT as basilar perivascular GGO, with fibrosis, traction bronchiectasis, and honeycombing resulting in architectural distortion. Desquamative interstitial pneumonia (DIP) is common in middle-aged male smokers [22]. Similar to COVID-19, DIP causes GGO with peripheral lower lobar predominance, but small cystic spaces may develop inside these GGO which is not a common finding in COVID-19 [23]. Unfortunately, organizing pneumonia has more similar CT features to COVID-19 pneumonia including the patchy GGO, consolidations, bronchovascular nodules, perivascular thickening, and reversed halo sign with bilateral lower lobar and subpleural predominance. Unlike COVID-19, the pulmonary consolidations in OP are more frequent and migratory with evident perilobular thickening [24].

In drug-induced lung injury, there is a history of specific drug intake (especially chemotherapeutic agents) and the presentation tends to be diffuse without site predilection or differentiating imaging features [25]. Pulmonary edema is a broad term describing the accumulation of fluids with the pulmonary extravascular spaces due to volume overload resulting from cardiac or non-cardiac conditions. Radiologically, there are perihilar GGO, consolidations, interstitial thickening, and pleural effusion [26]. In diffuse alveolar hemorrhage, the patients complain from recurrent hemoptysis as a result of bleeding into the alveolar spaces caused by various diseases such as coagulation disorders, vasculitides, and connective tissue diseases. In chest CT, there are widespread migratory GGO, consolidations with crazy-paving appearance [27].

Hypersensitivity pneumonitis results from long-time inhalation of an external allergen which promotes pulmonary immunological response that has different stages. In the acute stage, there is bilateral patchy GGO pattern, and in the subacute stage, there are centrilobular nodules and GGO with mosaic attenuation pattern, while in the chronic stage, there is bilateral midzonal perihilar fibrosis [28]. Eosinophilic pneumonia is often common in asthma patients; they show multiple GGO and consolidation with slight peripheral upper lobar predominance and possible crazy-paving appearance resulting from eosinophilic-rich infiltrate filling the pulmonary alveoli [29].

Clinically in our cohort, we found that fever (90%) and gastrointestinal symptoms (18.75%) were statistically more common (*p* < 0.001) in COVID-19 patients. Many studies focused on the detection of fever considering it one of the initial and cardinal signs in COVID-19 infection that can be correlated with the severity and progression of lung involvement as well as the adverse outcome of the disease [9, 13]. While gastrointestinal symptoms (including diarrhea, nausea, and vomiting) had less incidence in both groups, although they were statistically more common (*p* < 0.001) in COVID-19 group than the other non-COVID 19 group, this can be explained by the fact the novel coronavirus has the unique ability to bind with the ACE 2 receptors scattered along the gastric mucosa, resulting in non-specific gastritis and enteritis with subsequent electrolyte disturbances [30]. These changes are usually linked to severe/critical forms of COVID-19 showing higher grades of fever and serious constitutional symptoms (fatigue, headache, and breathlessness) [31].

One of the most pronounced differences between the two groups in the current study was the lymphopenia in COVID-19 patients (55%) which were statistically more common (*p* < 0.001) than the other group. The relation between COVID-19 and complete blood count changes is still controversial. In the cohort study conducted by Bai et al. [16], they found that patients with COVID-19 were more likely to have reduced leucocytic and lymphocytic count than patients with the non-COVID-19 illness. In another study conducted by Zheng et al. [32], they investigated 88 cases of COVID-19 and 22 cases of non-COVID-19 pneumonia, and they reported that lymphocytopenia is noticeable only in moderate and severe cases of COVID 19 patients and it could be a critical indicator for the clinical deterioration not only a consequence of the viral infection. By contrast, in a small-sized cohort study conducted by Xiong et al. [33], they reported that abnormally reduced peripheral blood count was only detected in few of COVID 19 cases included in their study and the majority of cases had normal white blood cell count, neutrophil count, and lymphocyte count.

This study has several limitations; first, the experience level of the assigned radiologist was more than 10 years and inclusion of radiologist with less experience or general radiology training not specific to diagnose chest scans may have changed the diagnostic outcomes. We believe that general radiologists share the responsibility of COVID-19 diagnosis and differentiating it from other conditions especially at this point where we face a shortage of specialist radiologist with dedicated chest imaging training to interpret the massive number of chest CT scans done for suspected patients. The small size for this study population is another limitation and it remains indefinite if would improve in a more well-balanced and larger-scale prospective study of similar design. Finally, the assigned radiologists were given limited clinical information during the assessment. The history of co-existing other morbidities such as collagen diseases, autoimmune diseases, or cardiac conditions as well as exposure history to aerosolized antigens or drug intake was not disclosed during evaluation. Furthermore, data about the onset of respiratory manifestation like cough and dyspnea as well as history of drug intake, exposure to aerosolized antigen, or co-existing morbidities was not available during evaluation, which could have further enhanced the diagnostic performance.

## Conclusion

In this cohort, chest CT offered reasonable sensitivity in differentiating COVID-19- from non-COVID-19-associated GGO with low specificity in differentiating COVID-19 from other viral pneumonias and moderate specificity in differentiating COVID-19 from other causes of GGO. So, multidisciplinary approach including detailed radiological assessment, exact clinical scenarios, and assisting laboratory data can help to reach an accurate diagnosis and reduce the number of CT false-positive cases especially in times of pandemics.

## Data Availability

All data generated or analyzed during this study are included in this published article.

## Abbreviations

COVID-19: Coronavirus disease 2019
GGO: Ground-glass opacity
RT-PCR: Reverse transcription-polymerase chain reaction
BAL: Bronchoalveolar lavage
SARS: Severe acute respiratory syndrome
MERS: Middle East respiratory syndrome
PJP: *Pneumocystis jiroveci* pneumonia
NSIP: Non-specific interstitial pneumonia
DIP: Desquamative interstitial pneumonia
COP: Cryptogenic organizing pneumonia
HP: Hypersensitivity pneumonia
EP: Eosinophilia pneumonia
DAH: Diffuse alveolar hemorrhage
